# Genetic and systems level analysis of *Drosophila sticky/citron kinase *and *dFmr1 *mutants reveals common regulation of genetic networks

**DOI:** 10.1186/1752-0509-2-101

**Published:** 2008-11-25

**Authors:** Christopher R Bauer, Andrew M Epstein, Sarah J Sweeney, Daniela C Zarnescu, Giovanni Bosco

**Affiliations:** 1 Department of Molecular and Cellular Biology, University of Arizona, Tucson, USA

## Abstract

**Background:**

In *Drosophila*, the genes *sticky *and *dFmr1 *have both been shown to regulate cytoskeletal dynamics and chromatin structure. These genes also genetically interact with Argonaute family microRNA regulators. Furthermore, in mammalian systems, both genes have been implicated in neuronal development. Given these genetic and functional similarities, we tested *Drosophila sticky *and *dFmr1 *for a genetic interaction and measured whole genome expression in both mutants to assess similarities in gene regulation.

**Results:**

We found that *sticky *mutations can dominantly suppress a *dFmr1 *gain-of-function phenotype in the developing eye, while phenotypes produced by RNAi knock-down of *sticky *were enhanced by *dFmr1 *RNAi and a *dFmr1 *loss-of-function mutation. We also identified a large number of transcripts that were misexpressed in both mutants suggesting that *sticky *and *dFmr1 *gene products similarly regulate gene expression. By integrating gene expression data with a protein-protein interaction network, we found that mutations in *sticky *and *dFmr1 *resulted in misexpression of common gene networks, and consequently predicted additional specific phenotypes previously not known to be associated with either gene. Further phenotypic analyses validated these predictions.

**Conclusion:**

These findings establish a functional link between two previously unrelated genes. Microarray analysis indicates that *sticky *and *dFmr1 *are both required for regulation of many developmental genes in a variety of cell types. The diversity of transcripts regulated by these two genes suggests a clear cause of the pleiotropy that *sticky *and *dFmr1 *mutants display and provides many novel, testable hypotheses about the functions of these genes. As both of these genes are implicated in the development and function of the mammalian brain, these results have relevance to human health as well as to understanding more general biological processes.

## Background

In multicellular organisms, developmental processes must coordinate cytoskeletal dynamics and morphogenesis with cell proliferation. For example, microtubule mediated trafficking and cell motility are inhibited during mitosis since centrosome and mitotic spindle mediated chromosome segregation requires dramatic reorganization of microtubules. In addition, actin/myosin mediated cell migration ceases during cell division when cortical actin must be reorganized in order to specify where the cleavage furrow is activated during cytokinesis. Consequently, gene expression patterns that control differentiation, morphogenesis and cell division must respond to a myriad of developmental cues and integrate them into a concerted cellular response in order to achieve proper tissue size and shape (for review see [1]).

It is also thought that changes in chromatin structure accompany developmental changes in order to establish and/or maintain tissue specific gene expression states [2,3]. How changes in chromatin are coordinated with cell division and cell differentiation remains poorly understood. However, it is clear that these processes must be linked in order to ensure accurate propagation of epigenetic states and maintenance of cell fates [2-4]. As these are fundamental processes ubiquitous to all metazoans, it is of great interest to uncover the factors that link cell cycle progression, differentiation and morphogenesis to developmental changes in chromatin.

*Drosophila *development, and oogenesis in particular, has proven to be an excellent model for understanding how developmental cues coordinate differentiation with cell cycle progression [1,5,6]. The *Drosophila *model has recently been used to reveal novel epigenetic functions of *sticky *and the *Fragile-X mental retardation-1 *(*dFmr1*) gene [7,8]. Furthermore, both genes have been shown to regulate actin/myosin cytoskeletal organization and mutants in both genes exhibit pleiotropy. These findings suggest that *sticky *and *dFmr1 *may possess similar biological functions and may represent regulatory hubs that coordinate diverse cellular and developmental processes. If this is true, it would be expected that both of these genes would have many interactors as it has been suggested that pleiotropy may result from disrupting hubs in protein-protein interaction networks as well as in gene regulatory networks [9,10].

*sticky *is the *Drosophila *homologue of mammalian *citron kinase*. It is related to protein kinase B, protein kinase C, Rho-Kinase (ROK), myotonic dystrophy protein kinase (DMPK) and myotonin-related Cdc42-binding kinase (MRCK) [11,12] (for review see [13]). The only known substrate for citron kinase is myosin II, the primary motor protein responsible for cleavage furrow ingression during cytokinesis [14]). Although it is clear that *sticky/citron kinase *plays a critical function in cytokinesis, this kinase has other functions as it likely has more than one substrate. In addition, it may possess activities not dependent upon its kinase domain. *Citron kinase *deficient mice exhibit cytokinesis failure and increased apoptosis in the central nervous system resulting in severe ataxia and epilepsy. However, some non-neuronal cells develop normally [15-18]. Normal development of non-neuronal cells suggests that, in mice, *citron kinase *has a neuron specific function. In a Down syndrome mouse model *citron kinase *is responsible for inhibiting neurite extension [19]. Interestingly, this *citron kinase *mediated neurite inhibition is through a direct interaction with tetratricopeptide repeat protein TTC3, the ortholog of *Drosophila dTPR2*, which suppresses polyglutamine toxicity in a fly Huntington's disease model [19,20]. Further support for a neuro-specific function comes from the observation that a splice variant of citron kinase protein, which lacks the kinase domain, directly interacts with post-synaptic density protein 95 (PSD95) and localizes to synapses [21-24].

The Fragile-X mental retardation-1 protein (FMRP) is a microRNA regulator, and like citron kinase protein, FMRP is thought to have critical functions in neurodevelopment [25]. Inactivation of the human *FMR1 *ortholog leads to Fragile-X syndrome, which is the most common form of inherited mental retardation and is associated with severe neurodevelopmental and behavioral abnormalities (for review see [26]). FMRP is an RNA binding protein, and its molecular function is thought to be in regulating mRNA translation and mRNA trafficking in neurons. Interestingly, in *Drosophila, dFmr1 *has also been shown to regulate heterochromatin mediated gene silencing [7]. Therefore, it is clear that Fmrp is capable of affecting the expression of many genes, some of which produce mRNA molecules that are direct binding targets of the Fmrp protein while others may be targets of Fmrp chromatin mediated epigenetic gene regulation [7,25]. Both *sticky *and *dFmr1 *have been shown to genetically interact with the microRNA regulator *Ago1*, further supporting a connection between these genes in translational and epigenetic control of gene expression [8,27].

Fmrp also regulates actin/myosin cytoskeletal dynamics through the Rac1 GTPase [7,28-31]. Similarly, in both *Drosophila *and mouse, *sticky *(*citron kinase*) is regulated by Rho and Rac GTPase activities [12,32]. This raises the interesting possibility that both citron kinase protein and Fmrp could regulate actin/myosin cytoskeletal dynamics *via *a common signaling pathway. Given the phenotypic and genetic similarities between the *Drosophila sticky *and *dFmr1 *genes, we first investigated whether mutations in these two genes exhibited a genetic interaction. Secondly, we assessed whether both mutants had a common suite of genes that were misregulated that could account for the similarities between *sticky *and *dFmr1 *mutant phenotypes.

In this report, we show that the *Drosophila citron kinase *ortholog, *sticky*, exhibits a genetic interaction with *dFmr1*, and we show that *sticky *and *dFmr1 *regulate a common set of genes. These findings have important implications for the functions of both citron kinase protein and FMRP in human neurodevelopment as well as human pathologies including Down syndrome and Fragile-X syndrome.

## Results and discussion

### *sticky *mutants dominantly suppress *dFmr1 *gain of function rough eye phenotype

**Figure 1:**
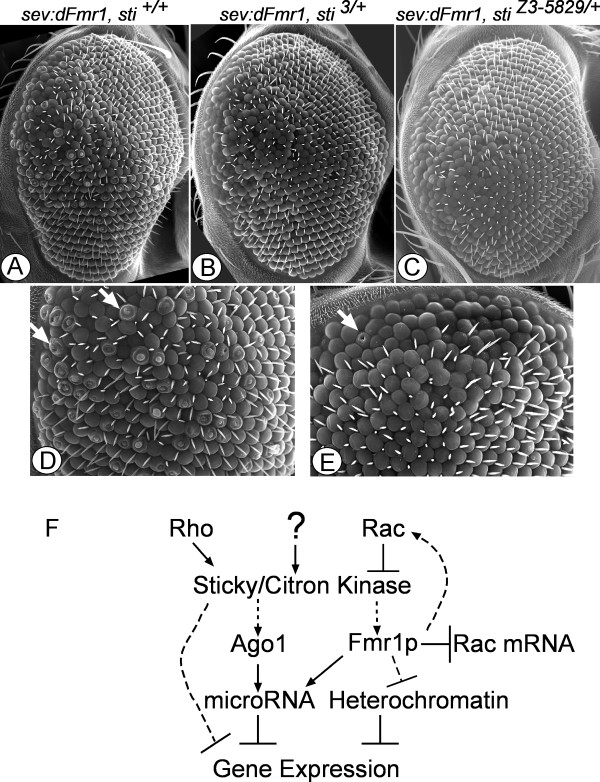
***sticky *mutations rescue *dFmr1 *overexpression phenotypes in the *Drosophila *eye**. (A) Scanning electron microscope visualization of eye developmental defects due to overexpression of *dFmr1 *by the *sevenless *promoter in a *sticky *wild-type background (*sev:dFmr1, sti*^+/+^). (B-C) One mutant copy of the *sticky*, *sti*^3/+ ^or *sti*^*Z*3-5829/+ ^dominantly suppresses the rough eye phenotype caused by *sev:dFmr1 *overexpression. (D-E) The crater-like necrotic adult eye tissue caused by the *sev:dFmr1 *overexpression (see arrows) is almost completely suppressed by one mutant copy of *sti*^*Z*3-5829/+^. (F) Rho is known to directly activate *sticky *(solid arrow) and Rac is known to inhibit (solid bar) *sticky *activity. Other, unknown (?) developmental and cellular signals may also regulate *sticky *activity. We propose that both Ago1 and Fmrp activities are downstream of *sticky*, and that some wild-type or gain-of-function Ago1 and Fmrp dependant processes are sensitive to *sticky*. Dashed arrows indicate possible indirect interactions. This model is supported by the genetic interaction between *sticky *and *dFmr1 *shown above and that previously reported for *sticky *and *Ago1 *[8]. Both Ago1 and Fmrp are known to directly control microRNA mediated gene regulation (solid arrows). Fmrp also directly targets Rac1 mRNA for repression [28]. *sticky *and Fmrp proteins also control gene expression through heterochromatin gene silencing (bars and dashed arrows). It is not known whether these effects on heterochromatin are direct or indirect.

We have previously shown that an *Argonaute1 *(*Ago1*) gain of function phenotype can be dominantly suppressed by loss-of-function mutations in *sticky *[8]. This observation suggested that wild-type Ago1 protein function was dependent on proper *sticky *dosage (Figure 1F). Fmrp, like Ago1, is a regulator of microRNA mediated gene silencing [25]. In addition, *Drosophila dFmr1 *mutants have been shown to have heterochromatin structure and gene silencing defects similar to those reported for *sticky *mutants [7,8]. Therefore, we tested whether *sticky *genetically interacts with *dFmr1*.

Two different *sticky *alleles, *sti*^3 ^and *sti*^*Z*3-5829^, were crossed to a *sev:dFmr1 *strain where *dFmr1 *is being overexpressed specifically in the eye under the *sevenless *gene enhancer [33]. As previously reported the *sev:dFmr1 *eyes were rough (Fig. 1A). At higher magnification the *sev:dFmr1 *eye showed that 100% (n = 10 eyes) had >50 ommatidia with crater-like necrosis (see arrows in Fig. 1D). Both *sti*^3 ^and *sti*^*Z*3-5829 ^mutant alleles were able to dominantly suppress the *dFmr1 *gain-of-function rough eye phenotypes yielding a less severe rough eye (Fig. 1B and 1C). Although the degree of suppression was variable, it occurred in 100% of progeny (n>100). Furthermore, the crater-like necrosis of the individual ommatidia was almost completely suppressed as all (n = 10) eyes we observed at high magnification had <5 of these defects per eye (see arrows, Fig. 1D and 1E). We conclude that Fmrp function is sensitive to *sticky *gene dosage. This also suggests that, in part, Fmrp effector functions are *sticky *dependent, and therefore we propose that *sticky *may function upstream of Fmrp (Fig. 1F). However, since the Rac-GTPase may be positively or negatively regulated by Fmrp [28,29], it is also possible that Fmrp activity modulates *sticky *through Rac activation or Rac mRNA translational repression (Fig. 1F).

### Loss of *dFmr1 *function enhances *sticky *knockdown phenotypes in the eye

**Figure 2:**
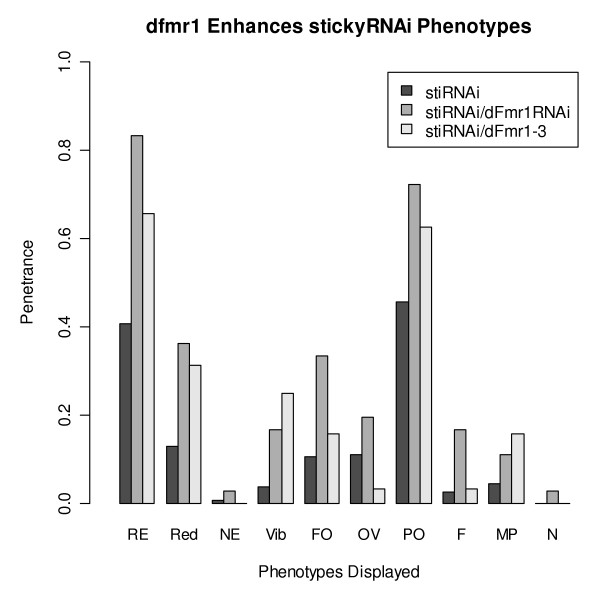
**dFmr1 loss of function enhances sticky RNAi knockdown phenotypes**. Plot of phenotype penetrance for the genotypes: *ey-GAL4*/+;*UAS-sticky *RNAi/+ (n = 162), *ey-GAL4/UAS-dFmr1 *RNAi; *UAS-sticky *RNAi/+ (n = 36), and *ey-GAL4*/+;*UAS-sticky *RNAi/*dFmr1*^3 ^(n = 32). No phenotypes were observed in flies of the genotype *ey-GAL4/UAS-dFmr *RNAi (data not shown). Phenotypes included are Rough Eye (RE), Reduced Eye (Red), No Eye (NE), Vibrissae (Vib), Frontorbital bristles (FO), Orbital and Vertical bristles (OV), Postorbital bristles (PO), Frontal bristles (F), Maxillary Palps (MP), and Necrosis (N). For RE, Red, Vib and PO we found statistically significant enhancement in the double RNAi lines when compared to the *sticky*-RNAi line alone, *p *< 0.05 in a Fisher's Exact test. This was also true for *sticky*-RNAi line in a *dFmr1*^3 ^heterozygous compared to the *sticky*-RNAi line alone.

The ability of *sticky *mutations to rescue *dFmr1 *overexpression suggests positive regulation between these genes. In a model such as this (Fig. 1F), it would be predicted that a loss of function in both genes would result in phenotype enhancement. It has previously been shown that RNAi knockdown of *sticky*, under control of the eyeless promoter, results in a rough eye phenotype and a reduction in eye size [12]. We have used this system to demonstrate that loss of function in *dFmr1 *can enhance the phenotypes caused by *sticky *RNAi knockdown in the fly eye (Fig. 2).

Flies containing an *eyeless-GAL4 *driver were crossed to a line containing a UAS-*sticky*-RNAi construct. This resulted in rough and reduced eyes in many of the progeny. We also observed a strong effect on the bristles that border the eye as well as a variety of low frequency phenotypes including complete loss of the eye, reduced or missing maxillary palps, and necrotic ommatidia. We crossed the *ey-GAL4*; *UAS-sticky*-RNAi lines to *UAS-dFmr1*-RNAi lines and to *dFmr1*^3 ^mutants. In both cases, reducing the function of *dFmr1 *enhanced the phenotypes caused by *sticky *RNAi knockdown. In the cases of rough eyes, reduced eyes, vibrissae, and postorbital bristles, the phenotypes caused by sticky knockdown were significantly enhanced by both *dFmr1 *knockdown and a *dFmr1*^3 ^loss of function allele (p < .05, Fisher's Exact test). When the *ey-GAL4 *driver was used to express the *dFmr1 *RNAi construct alone, no phenotypes were observed (n = 62).

### *sticky *and *dFmr1 *regulate a common set of genes

*sticky *and *dFmr1 *genetically interact and share a common set of genetic interactors such as *Ago1*, *Rac*-GTPase and possibly others (Fig. 1F). Since mutations in the *sticky *and *dFmr1 *genes have been demonstrated to affect chromatin structure, we also hypothesized that regulation of gene expression by sticky kinase and/or Fmrp could occur at the level of transcription and therefore mRNA levels would be altered in each of these mutants. To assess which specific transcripts were being regulated by these genes, we used NimbleGen *Drosophila *whole genome arrays containing ~385,000 features (12 unique features per gene, synthesized in duplicate on each array) to measure the levels of 15,634 specific transcripts in Cy3 labeled cDNA made from whole female fly total RNA. Due to the number of measurements per gene, we were able to identify a large set of misexpressed genes with high significance.

**Figure 3:**
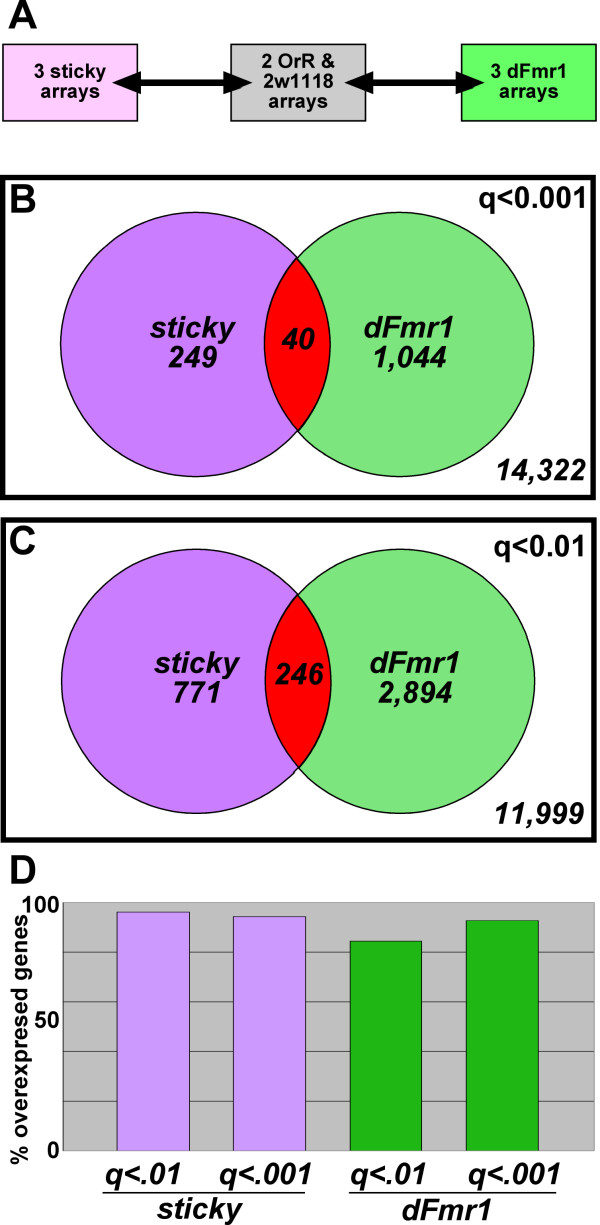
**A common set of genes is misexpressed in *sticky *and *dFmr1 *mutants**. (A) Three biological replicates of *sticky *total RNA and three biological replicates of *dFmr1 *mutants were analyzed by NimbleGen gene expression arrays. These data were compared to expression data from two biological replicates of *OrR *and two biological replicates of *w*^1118 ^wild-type control RNA. Expression data from the two controls were combined in order to better account for differences in genetic background. (B-C) Venn diagrams showing the number of genes with significantly altered expression levels in *sticky *and *dFmr1 *mutants. (B) With a significance threshold of q<.001, we found 249 misexpressed genes and 1,044 misexpressed genes in *sticky *and *dFmr1 *mutants, respectively. There were 40 genes differentially expressed in both mutant strains (p = 2.37e-6) and 14,322 genes with no significant change in expression. (C) With a threshold of q<.01, we found 771 misexpressed genes and 2,894 misexpressed genes in *sticky *and *dFmr1 *mutants, respectively. There were 246 genes differentially expressed in both mutants (p = 4.27e-14) and 11,999 genes with no significant change in expression. (D) Of the genes that were misexpressed in either mutant the vast majority of them were overexpressed.

Expression array data obtained from three biological replicates of each mutant were compared to a pooled set of two biological replicates for each of two independent wild-type controls (Fig. 3A). One wild-type control was *Oregon-R*. The second wild-type control was from a stock made in a *w*^1118 ^background, homozygous for *dFmr1*^3^, and also carried a fully functional wild-type *dFmr1 *transgene that rescued the *dFmr1*^3 ^mutant phenotypes. Therefore, this second control stock had a genetic background identical to that of the *dFmr1*^3 ^mutant. This served to reduce the chance of identifying genes which showed different transcript abundance due to genetic background differences. The use of two independent wild type controls also served to reduce the chance of detecting transcripts which may have large natural variances in abundance but do not dramatically impact phenotype. Adult female flies from control stocks were grown and aged under identical conditions as the mutants.

**Figure 4:**
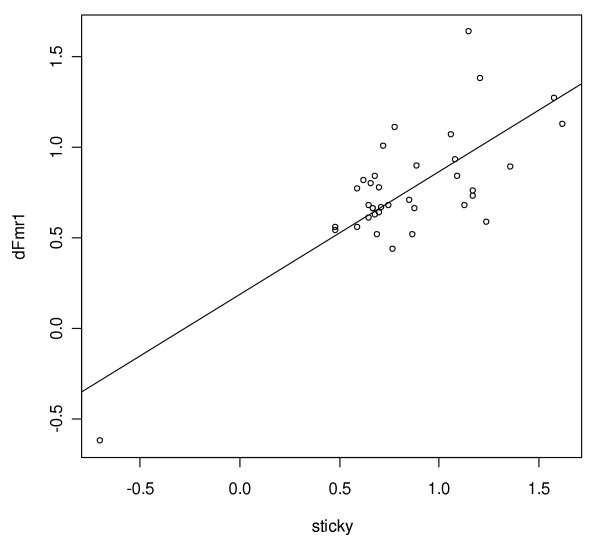
**Commonly misexpressed genes tend to be overexpressed**. Plot of microarray log fold changes observed in *sticky *versus *dFmr1 *for the 40 genes found to be differentially expressed in both mutants. R^2 ^= .581 and a two-tailed P value is < 0.0001 for 39 degrees of freedom.

We observed that *sti*^3^/*sti*^*Z*3-5829 ^females had 249 genes and 771 genes misexpressed with a false discovery rate of q < 0.001 and q < 0.01, respectively (Fig. 3 and Additional File 1). Similarly, *dFmr1*^3^/*dFmr1*^3 ^females had 1,044 and 2,894 genes misexpressed with q < 0.001 and q < 0.01, respectively (Fig. 3 and Additional File 2). Strikingly, a comparison of these two sets revealed a highly significant overlap (Fig. 3). With the more stringent q-value threshold, 40 genes were found to be differentially expressed on both lists. This is significantly greater than the number one would expect to find by chance (p = 1.91 × 10^-7^, see methods). Each of these 40 genes showed a change in the same direction in both mutants (Fig. 4). With the more relaxed threshold of q < 0.01, 246 genes are common to both lists (p = 3.23 × 10^-20^) with 227 of these genes (~92%) commonly up-regulated in the mutants (Additional File 3).

**Figure 5:**
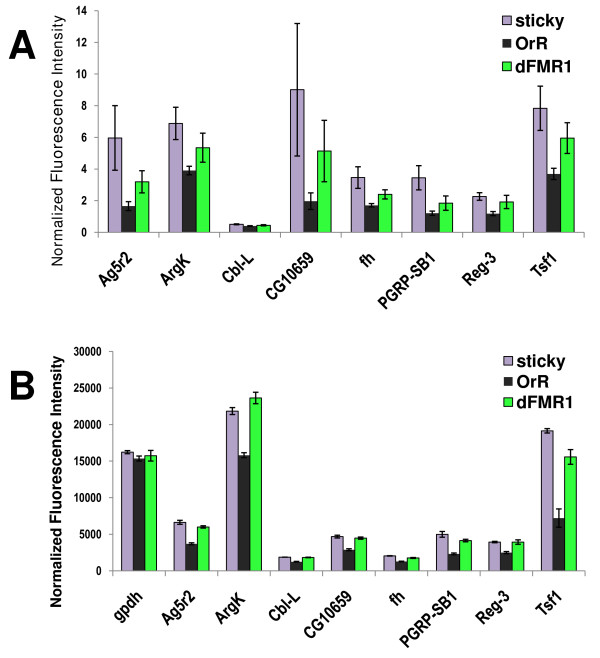
**RT-PCR quantitation of mRNA levels for candidate genes**. (A) Mean gene expression levels with standard errors as measured by RT-PCR. For each gene, band intensity was normalized to *gpdh *expression from the same cDNA template. (B) Mean gene expression levels with standard errors as measure by microarray.

To assess the validity of these findings we used semi-quantitative RT-PCR to measure the abundance of 8 different transcripts that were enriched in both mutants. We confirmed that 8 of 8 genes are indeed being overexpressed in *sticky *mutants and 7 of 8 genes were upregulated in *dFmr1 *mutants (Fig. 5). Though the *Cbl*-L transcript signal appeared to be higher in the *dFmr1 *mutant than in the wild-type control, we could not conclude that this difference was statistically significant. This may be because *Cbl*-L is expressed at very low levels in all genotypes and our assay was simply not sensitive enough to detect such a small difference. Regardless, this RT-PCR validation provides general evidence that *sticky *and *dFmr1 *both serve to normally repress a common set of genes, and these data suggest that a large number of similar processes may be affected by both *sticky *and *dFmr1*.

### sticky kinase and Fmrp regulate a common set of biological processes

**Figure 6:**
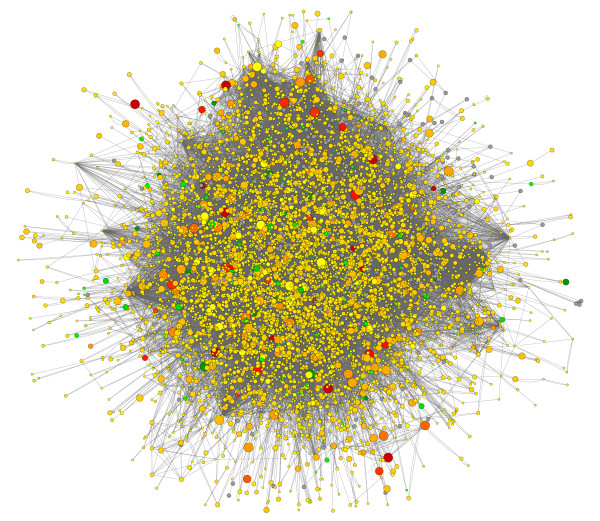
**Expression Network**. Protein-protein interaction network overlaid with *sticky *mutant expression data. Red indicates genes that are up-regulated in *sticky *mutant compared to wild-type and green indicates down-regulation. Yellow nodes represent genes with little to no change in expression and gray nodes represent genes that could not be assigned expression data. Node size indicates the significance (i.e. p-value) of the change in expression, where larger nodes are more significant and smaller nodes are less significant.

Though the analyses described above demonstrate that many genes are commonly regulated by sticky kinase and Fmrp with a high degree of statistical significance, the biological significance of this regulation was not evident from the gene lists alone. To overcome this problem we associated our expression data with a network of *Drosophila *protein-protein interactions. Transcriptional regulation is only one of many levels of regulation found in biology. Though microarrays do not directly provide data on other types of regulation, the effects of transcriptional changes on other levels of biological regulation can be inferred based on additional information. For example, if a gene does not show a change in transcript abundance but nevertheless interacts with a number of genes that show significant expression changes, it is likely that its functionality will be altered. Thus, we used protein-protein interaction data to expand the scope of our microarray analysis. After associating our microarray gene expression data set with a protein-protein interaction network (Fig. 6), we used jActiveModules to identify and score sub-networks of interacting genes that collectively showed significant changes in expression [34].

**Figure 7:**
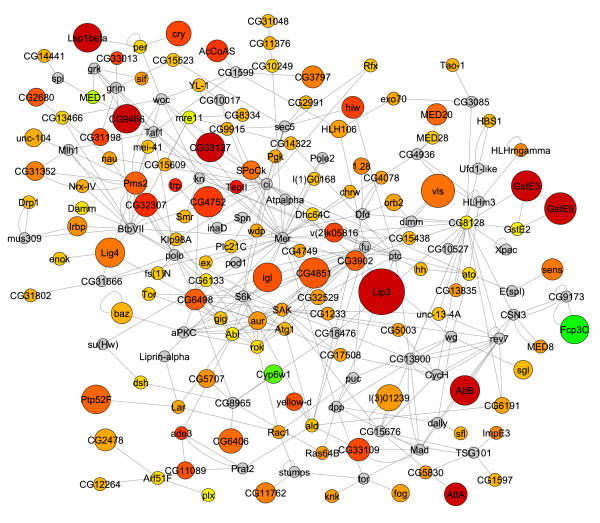
***sticky *mutant active sub-network**. Highest scoring sub-network found by jActiveModules using *sticky *mutant expression data. Red indicates genes that are up-regulated in the *sticky *mutant compared to wild type and green indicates down-regulation. Yellow nodes represent genes with little to no change in expression and gray nodes represent genes that could not be assigned expression data. Node size indicates the significance (i.e. p-value) of the change in expression, where larger nodes are more significant and smaller nodes are less significant.

**Figure 8:**
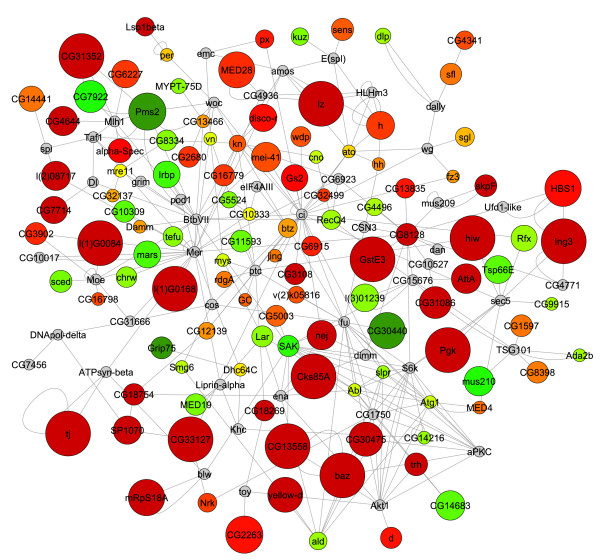
***dFmr1 *mutant active sub-network**. Highest scoring sub-network found by jActiveModules using *dFmr1 *expression data. Red indicates genes that are up-regulated in *dFmr1 *compared to wild type and green indicates down-regulation. Yellow nodes represent genes with little to no change in expression and gray nodes represent genes that could not be assigned expression data. Node size indicates the significance (i.e. p-value) of the change in expression, where larger nodes are more significant and smaller nodes are less significant.

For each mutant expression data set the highest scoring sub-networks revealed several interesting features (Fig. 7 and 8). First, we observed that for both mutants, the *Mer*, *ci *and *aPKC *genes emerge as common hubs for the two sub-networks even though none of these genes are being misexpressed in either mutant (gray nodes in Fig. 7 and 8). As noted above, this is due to many of the same genes that are being misexpressed in both mutants. Second, we found that although both *sticky *and *dFmr1 *mutants generally tend to overexpress genes (Fig. 4), the *dFmr1 *sub-network shows many more underexpressed genes than the *sticky *sub-network (Fig. 7 and 8, green nodes). Third, although the *dFmr1 *sub-network shows many more underexpressed genes than the *sticky *sub-network these are not necessarily genes that are misregulated only in the *dFmr1 *mutant. For example, the DNA repair genes, *Irbp *and *Pms2*, are overexpressed in *sticky *mutants whereas both are underexpressed in *dFmr1 *mutants (Fig. 7 and 8). Similarly, the Autophagy-specific gene 1 kinase, *Atg1*, gene is overexpressed in *sticky *mutants whereas it is underexpressed in *dFmr1 *mutants (Fig. 7 and 8). Lastly, although DNA damage response and repair genes are common in both sub-networks, we noted that different subsets of these genes are misexpressed in each of the two mutants. For example, the *Lig4 *and *mus309 *genes are present in the *sticky *sub-network but not the *dFmr1 *sub-network, whereas the *RecQ*, *mus209 *and *mus210 *genes emerge from the *dFmr1 *sub-network and not observed in the *sticky *sub-network. This suggests that although both mutants may impinge on similar biological processes by altering expression of similar sets of genes, the mechanism by which *sticky *and *dFmr1 *effect these same processes may be different. In the case of these DNA repair genes, it will be interesting to see whether these differences in the two sub-networks reflect physiologically different types of DNA damage and/or repair processes.

**Figure 9:**
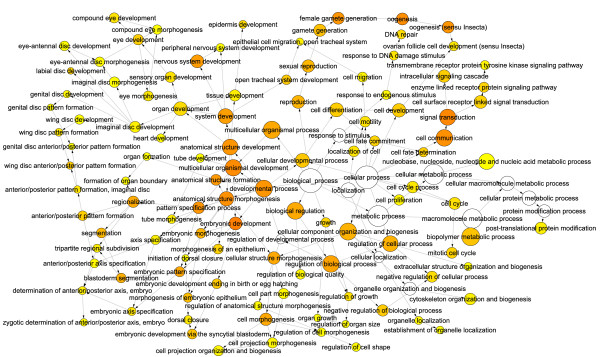
***sticky *BiNGO**. Directed acyclic graph of over-represented GO-terms in the highest scoring *sticky *active sub-network. Node size indicates the number of genes associated with each GO-term (i.e. larger nodes represent GO-terms associated with many genes in the active sub-network). Node color indicates significance of over-representation based on Benjamini-Hochberg corrected p-value: white = p > .0001, yellow = p < .0001, red = p << .0001.

**Figure 10:**
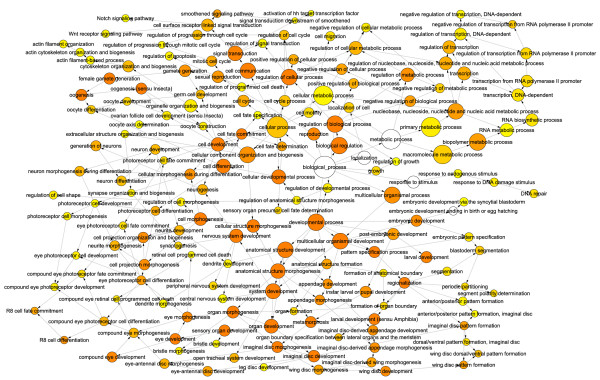
***dFmr1 *BiNGO**. Directed acyclic graph of over-represented GO-terms in the highest scoring *dFmr1 *active sub-network. Node size indicates the number of genes associated with each GO-term (i.e. larger nodes represent GO-terms associated with many genes in the active sub-network). Node color indicates significance of over-representation based on Benjamini-Hochberg corrected p-value: white = p > .0001, yellow = p < .0001, red = p << .0001.

As a quantitative measurement, we used the genes within these sub-networks to identify significantly overrepresented gene ontology terms (GO-terms). Several common GO-terms were overrepresented in the highest scoring sub-networks for both *sticky *and *dFmr1 *mutant gene expression patterns. These GO-terms included many related to embryonic and larval development, nervous system development, oogenesis, cytoskeletal organization, axis specification, cell cycle regulation, and DNA damage responses (Fig. 9 and 10). Many of these processes fit well with our current understanding of the *in vivo *functions of these two genes yet some suggest novel functions. For example, according to our analysis "cytoskeleton organization and biogenesis" is altered in both *sticky *and *dFmr1 *mutants. This is consistent with previous reports in the literature. Intriguingly, our GO-term analysis predicts disruptions in oocyte and embryonic axis specification and DNA repair, two processes that have not been reported to be affected in either *sticky *or *dFmr1 *mutants.

As a control for this analysis, we compared gene expression in our two different wild-type control strains. We observed 191 and 365 genes (q<.001 and q<.01, respectively) to be differentially expressed between these two strains. These expression changes were integrated with our protein-protein interaction network and used to identify significantly altered genetic modules. The highest scoring sub-network did not possess any informative GO-terms which were significantly over-represented (data not shown).

As a further test of the significance of this GO-term analysis, we randomized the microarray data sets by randomly reassigning gene names to the microarray gene expression p-values (see methods), and we asked whether these would yield any specifically enriched GO-terms that were also found in the non-randomized data set. We found that the *sticky *mutant randomized expression data set produced no significantly enriched GO-terms (data not shown). By contrast, the *dFmr1 *mutant randomized data set did yield a small number of significantly enriched GO-terms, such as "RNA-pol II transcription" and "transcriptional regulation" (data not shown). This may represent bias that is inherent to our protein-protein interaction network.

### *sticky *and *dFmr1 *regulate genes with similar tissue specificity

The GO-terms found to be over-represented in the identified sub-networks (Fig. 9 and 10) suggested some misexpression may have been tissue specific. However, since our RNA was prepared from whole adult flies, it was not possible to directly determine which tissues were misexpressing which genes. In order to address this issue we compared our data to the FlyAtlas dataset [35]. This data set compares gene expression profiles between individual *Drosophila *tissues. However, the criteria used to define tissue specific genes in Chintapalli et al. were as strict as possible (no detectable signal in more than one tissue) and resulted in small lists of tissue specific genes. We wished to determine larger sets of "tissue enriched" genes from FlyAtlas that could be more robustly compared to our microarray data. Therefore, we defined our own sets of "tissue enriched" genes (see methods) based on the ratio of gene expression in a given tissue to that of the whole fly, as reported in [35]. We were then able to calculate the probability of observing a given amount of overlap between sets.

**Figure 11:**
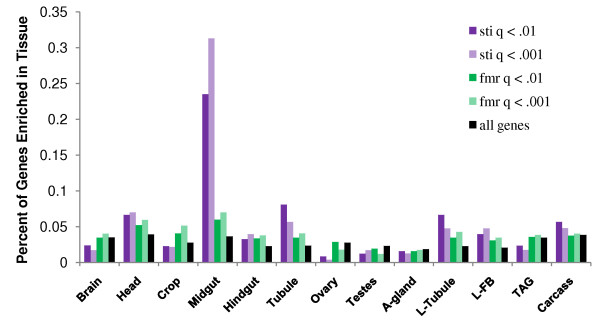
**Tissue specificity of misexpressed genes**. For each tissue (head, brain, crop, midgut, hindgut, Malpighian tubule, ovary, testes, accessory gland, larval Malpighian tubule, larval fat body, thoracico-abdominal ganglia, and thoracic and abdominal carcass), a list of genes enriched in that tissue was compiled based on the FlyAtlas data set (see methods). These lists were then compared to the lists of misexpressed genes in *sticky *and *dFmr1 *mutants at q<.01 and q<.001. Each column represents the percent of all misexpressed genes in a given list that were enriched in a given tissue. The black columns represent the percent of all genes in the fly genome that were defined as enriched in a particular tissue. These can be interpreted as the percentage of tissue enriched gene that would be expected in a random list of genes.

By comparing our misexpression lists with tissue enrichment lists derived from FlyAtlas, we observed both significant over- and under-representation of tissue enriched genes. We found that genes enriched in the head, midgut, malphigian tubules, larval tubules, and larval fatbody were significantly over-represented in the lists of misexpressed genes for both *sticky *and *dFmr1 *mutants (q<.001 and q<.01) (Fig. 11). Hindgut enriched genes were also highly represented on both lists though this was just shy of significance for *dFmr1 *misexpressed genes. We also found that genes enriched in ovaries and testes were under-represented in both the *sticky *and *dFmr1 *misexpression sets though the significance of this varied based on which q-value cutoff was used (Fig. 11). The under-representation of testes enriched genes was expected since we used female total RNA. However, under-representation of ovary specific genes was surprising since nearly half the RNA of mature adult females comes from ovaries, and this could have biased our analysis toward ovary enriched genes. Alternatively, *sticky *and *dFmr1 *could regulate genes that are not particularly abundant in ovaries but nevertheless have critical ovary specific functions, and therefore give rise to tissue specific phenotypes.

### *sticky *and *dFmr1 *mutants exhibit predicted oocyte polarity defect

In order to validate some GO-term analysis predictions we turned again to the *sticky *and *dFmr1 *mutants. GO-term analysis predicted that the *sticky *and *dFmr1 *mutants may exhibit an oocyte axis polarity defect (Fig. 7 and 8). This hypothesis was further supported by the common misexpression of the *Cbl *gene (Table 1 and Figure 5), which encodes for a receptor tyrosine kinase-associated, E3 ubiquitin ligase [36]. The Cbl protein attenuates EGFR signaling, which is a major receptor tyrosine kinase signaling pathway in the developing ovary [36,37]. Furthermore, Fmrp has been shown to directly bind the *Cbl *mRNA, possibly regulating its stability and translation, thus adding an additional level of regulation to Cbl gene expression [33]. Disruption of EGFR signaling during oogenesis can lead to oocyte polarity defects [38], and thus overexpression of *Cbl *could potentially lead to polarity defects by inappropriately dampening EGFR signaling [36]. Alternatively, *sticky *is known to phosphorylate the *Drosophila *myosin II light chain, *spaghetti squash *(*sqh*), in order to promote cytokinesis [39]. Although genetic mosaic studies have demonstrated that loss of *sqh *function leads to oocyte dorsal/ventral axis polarity defects [40,41], it is not known whether sticky kinase is the effector kinase that regulates myosin II in a non-cytokinesis context such as oocyte axis determination.

**Table 1:** Genes similarly regulated by *sticky *and *dFmr1*

	***Sticky***		***dFmr1***	
**Gene Name**	**Log Fold Change**	**p-value**	**Log Fold Change**	**p-value**
Adh	0.65	1.62E-05	0.68	8.66E-06
*Ag5r2*	0.85	3.84E-08	0.71	7.76E-07
*antdh*	0.89	3.97E-05	0.90	3.34E-05
*Cbl*	0.59	4.90E-06	0.56	1.09E-05
*CG10659*	0.70	4.79E-06	0.64	1.74E-05
*CG10908*	0.48	3.98E-05	0.56	5.33E-06
*CG12656*	0.65	4.04E-05	0.61	9.80E-05
*CG13324*	1.08	2.56E-08	0.93	3.17E-07
*CG13325*	1.17	1.42E-08	0.76	1.40E-05
*CG13420*	0.70	4.44E-05	0.78	1.02E-05
*CG13482*	1.06	9.09E-09	1.07	7.54E-09
*CG15194*	0.88	1.05E-07	0.66	8.93E-06
*CG16904*	0.48	2.70E-05	0.54	6.41E-06
*CG16996*	0.59	4.88E-06	0.77	7.63E-08
*CG17012*	0.78	4.42E-05	1.11	2.52E-07
*CG17327*	0.69	6.19E-07	0.52	3.42E-05
*CG17352*	1.24	7.63E-11	0.59	2.49E-05
*CG31681*	1.36	4.07E-09	0.89	4.61E-06
*CG32642*	-0.70	8.48E-06	-0.62	4.01E-05
*CG4461*	0.68	1.34E-05	0.84	5.85E-07
*CG4653*	1.21	2.86E-11	1.38	1.92E-12
*CG4734*	1.62	9.69E-10	1.13	5.17E-07
*CG4847*	1.15	3.32E-05	1.64	1.82E-07
*CG5506*	1.13	1.66E-09	0.68	7.26E-06
*CG6432*	0.66	1.66E-05	0.80	1.08E-06
*CG8562*	0.68	4.98E-06	0.63	1.35E-05
*CG8774*	0.67	2.55E-05	0.66	2.96E-05
*CG9080*	0.62	3.00E-05	0.82	4.75E-07
*CG9396*	1.17	4.96E-09	0.73	1.13E-05
*CG9672*	0.72	1.30E-05	1.01	6.99E-08
*lambdaTry*	0.75	1.75E-06	0.68	6.62E-06
*lectin-37Db*	0.77	5.70E-09	0.44	3.34E-05
*mex1*	0.87	5.54E-08	0.52	9.37E-05
*PGRP-SB1*	1.09	1.77E-08	0.84	1.24E-06
*PGRP-SC1a*	0.71	1.16E-05	0.67	2.63E-05
*Reg-3*	0.67	2.46E-05	0.66	3.41E-05
*Tsf1*	1.58	1.46E-06	1.27	3.19E-05

**Figure 12:**
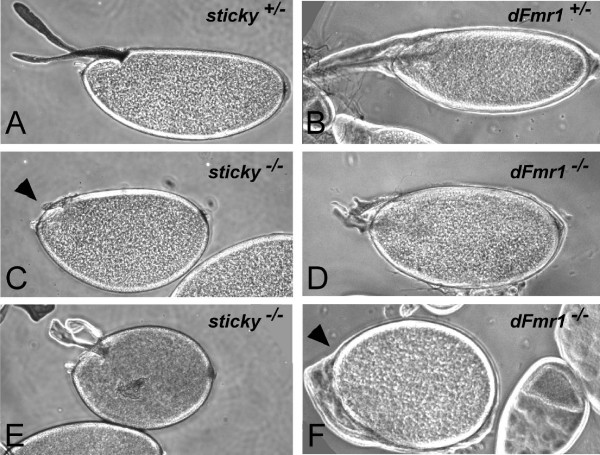
***sticky *and *dFmr1 *mutant oocytes exhibit predicted axis polarity defects**. (A-B) Phase contrast images of *sticky*^+/- ^wild-type and *dFmr1*^+/- ^wild-type, stage 14 oocytes showing normal dorsal/ventral axis formation as well as normal dorsal appendages. (C-E) Stage 14 *sticky*^*Z*3-5829^/*sticky*^3 ^(*sticky*^-/-^) and *dFmr1*^3/3 ^(*dFmr1*^-/-^) mutant oocytes are completely ventralized with missing or severely malformed dorsal appendages. Arrowheads point to regions of the oocytes where dorsal appendages would normally emanate.

We examined stage 14 oocytes in *sti*^3^/*sti*^*Z*3-5829 ^females and observed that 35% of oocytes were indeed ventralized, whereas the *sti*^3^/+ sibling heterozygous controls had normal oocytes (Fig. 1A,C,E). The variability in severity of oocyte ventralization is likely due to the fact that the *sti*^*Z*3-5829 ^mutation is not a complete loss-of-function [8]. Interestingly, the severity of this phenotype was temperature sensitive (data not shown), also consistent with our previous report demonstrating that the temperature sensitivity of the *sti*^*Z*3-5829 ^mutant allele [8]. Similarly, stage 14 oocytes from *dFmr1*^3^/*dFmr1*^3 ^mutant females were also severely ventralized, whereas a sibling heterozygous *dFmr1*^3^/+ had normal oocytes (Fig. 12B,D,F). This oocyte ventralization defect suggests that localization of axis determining factors requires sticky kinase and Fmrp function and/or that *Cbl *overexpression in the ovary can lead to polarity defects. In either case, we conclude that the polarity defects predicted by the GO-term analysis are valid, and this points to clear and testable mechanistic models of *sticky *and *dFmr1 *function in *Drosophila *oocyte axis formation.

## Conclusion

Mutations in *Drosophila sticky *and *dFmr1 *both result in a wide variety of phenotypes associated with many cell types and cellular processes. We believe many of these diverse phenotypes are caused by effects on the populations of a wide range of transcripts, either through transcriptional regulation or mRNA stability. Genes that function to regulate such a diverse array of processes can be resistant to characterization by traditional genetic methods. This is because pleiotropic effects make it difficult to infer function from phenotypes. Thus, we have used a combination of genomic and protein-protein interaction network analyses in order to compare the effects of mutations in *sticky *and *dFmr1*. We have found that these two genes function to negatively regulate a large number of common transcripts and that their targets are involved in a number of similar biological processes. It is noteworthy that this interpretation is also supported by previous reports that both sticky kinase and Fmrp are regulators of chromatin mediated epigenetic gene silencing. It has been shown that Fmrp is required for centric heterochromatin assembly during embryogenesis [7]. It has also been shown that *sticky *wild-type gene function is required for proper silencing of genes by heterochromatin [8]. Therefore, the finding that both mutants display overexpression of many genes is consistent with genetic evidence demonstrating their general gene silencing functions.

Based on our analyses, we predict that *sticky *and *dFmr1 *both function to control many aspects of development in imaginal disc derived appendages, oocytes, neurons, and possibly other tissues. Surprisingly, other biological processes that have not previously been associated with either gene are also predicted to be perturbed in *sticky *and *dFmr1 *mutants. For example, a number of genes functioning in DNA damage repair such as *DNA pol delta, Irbp, Ligase 4, mei-41, mre11, Mlh1, mus209, mus210, mus309, Pms2 *and *tefu*, among others, are revealed in the high scoring sub-networks of either one or both mutants (Fig. 9 and 10, Additional Files 1 and 2). We have validated at least one GO-analysis prediction by showing that *sticky *and *dFmr1 *mutants indeed exhibit an oocyte axis polarity defect (Fig. 12). Interestingly, *Drosophila *mutants in DNA repair functions, including *mei-41*, disrupt receptor tyrosine kinase (EGFR) signaling and results in oocyte polarity defects [42,43]. Therefore, it is possible that the *sticky *and *dFmr1 *mutant oocyte polarity defects could be due to failure to repair meiotic or germline chromosomal breaks.

Lastly, it is also noteworthy that the *Drosophila Merlin *(*Mer*) gene, the ortholog of the human tumor suppressor neurofibromatosis-2, emerged as a major hub for both *sticky *and *dFmr1 *active sub-networks (Fig. 7 and 8). This is important because the Mer protein serves to anchor actin to transmembrane proteins and is critical for establishing oocyte axis polarity [44]. It remains to be tested whether DNA damage repair, Cbl mediated attenuation of EGFR signaling, or actin/myosin defects is the primary cause of this axis polarity mutant phenotype. In addition, it remains to be tested whether other predictions from these analyses are correct.

Our claims of overlapping functions for the *sticky *and *dFmr1 *genes are supported by the demonstration of a genetic interaction and phenotypic similarities. The phenotypes that we observe in these mutants also serve to validate our computational predictions about the functions of these genes. Genes that regulate many biological processes also tend to exhibit pleiotropic effects, leading to multiple and seemingly unrelated phenotypes. As is the case with many other proteins that function as hubs within biological networks, understanding the molecular mechanisms through which sticky kinase and Fmrp affect any cellular process is complicated by the very fact that they impinge on many other regulatory molecules. The idea that mutations with pleiotropic effects are common to genes whose products have many protein-protein interactions is supported by a recent study of yeast pleiotropic genes [10]. Though few direct targets of Fmrp are known, and sticky kinase has only one known phosphorylation target, our expression data suggest these genes may nevertheless be hubs in the gene expression network (for reviews see, [13,45]). In addition, the great number of genes that are transcriptionally misregulated in both *dFmr1 *and *sticky *loss-of-function mutations could also explain pleiotropic effects [9]. Our systems level analysis of gene expression patterns in *sticky *and *dFmr1 *mutants has revealed gene networks, and potential direct downstream targets, that are regulated by both sticky kinase and Fmrp. It is our hope the future studies that seek to identify the molecular mechanisms causing the myriad phenotypes presented in these mutants may be facilitated by the gene networks reported in this study and the multiple testable hypotheses that arise from them, for example the predicted function of sticky kinase and Fmrp in cell polarity and DNA damage repair.

## Methods

### Drosophila strains

The *P w*^+ ^*[sev:dFmr1]*, CyO and *w*^1118^; +/+; *dFmr1*^3*TJ*^/*dFmr1*^3*TJ *^lines were previously described in [25]. The *dFmr1 *wild-type control used in the expression arrays has the genotype *w*^1118^; *P *[*w*+, *dFmr1*^+^*]/+; dFmr1*^3*TJ*^/*dFmr1*^3*TJ *^and was previously described [46,47]. This wild-type control, although homozygous mutant for the *dFmr1*^3*TJ*^, carries a fully functional transgene rescuing wild-type *dFmr1 *gene inserted into the *Drosophila *genome by P [*w+*] transposable element. Therefore, it is nearly genetically identical to the *w*^1118^; +/+; *dFmr1*^3*TJ*^/*dFmr1*^3*TJ *^mutant. The *sti*^3^/*TM6B Hu *and sti^*Z*3-5829^/*TM6B Hu *lines were previously described in [8].

### Genetic interaction tests

Female *sti*^3^/*TM6B Hu*^- ^and *sti*^*Z*3-5829^/*TM6B Hu*^- ^flies were crossed to males w/Y; *P w*^+ ^*[sev:dFmr1]*, CyO; +/+ carrying a *dFmr1 *eye-specific, overexpression transgene (*sev:dFmr1*). Adult progeny from these two crosses that were *P w*^+ ^*[sev:dFmr1]*, CyO;*sti*^-^/*Hu+ *(mutant *sticky*) and *P w*^+ ^*[sev:dFmr1]*, *CyO; sti*^+^/*TM6B Hu*^- ^(wild-type *sticky*) were scored for the rough eye phenotype caused by the Fmrp overexpression, as previously described [25]. Eyes were first scored under a dissecting light microscope, and adult flies were placed in 70% ethanol and stored up to one week. Samples were washed 5 times with 100% ethanol and dried under vacuum. Dried whole flies were coated with gold and imaged with an Electroscan E3 scanning electron microscope at 150–200× magnification. Images were captured directly as digital images.

For the *eyeless*-GAL4, double RNAi crosses, female *ey*-GAL4; UAS-*sticky*-RNAi/SM-TM6B flies were crossed to male UAS-*dFmr1*-RNAi/UAS-*dFmr1*-RNAi;+/+ flies. Females from the individual RNAi lines were also crossed to the *ey*-GAL4 stock males. Progeny of the appropriate genotypes were scored under a dissecting light microscope. For *ey*-GAL4/+; UAS-*sticky*-RNAi/+, n = 162. For *ey*-GAL4/UAS-*dFmr1*-RNAi; UAS-*sticky*-RNAi/+, n = 36. For *ey*-GAL4/+; UAS-*sticky*-RNAi/*dFmr1*^3^, n = 32. For *ey*-GAL4/UAS-*dFmr1*-RNAi, n = 62.

### RNA preparation and microarray statistics

We used NimbleGen *Drosophila *whole genome arrays containing ~385,000 unique features (12 features per gene, in duplicate) to measure the levels of 15,634 specific transcripts in Cy3 labeled cDNA made from the RNA of whole female flies fed on live yeast. RNA was purified using mirVana protocols from Ambion. Ten arrays were used to probe four different genotypes: Three arrays for *w*^1118^; +/+; *dFmr1*^3^/*dFmr1*^3^. Three arrays for +/+; +/+; *sti*^3^/*sti*^*Z*3-5829^. Two arrays for Oregon R-S wild-type control. Two arrays for *w*^1118^; *P *[*w*+, *dFmr1*^+^*]/+; dFmr1*^3^/*dFmr1*^3 ^transgene rescue strain control. Hybridizations, image analysis and quantile normalization were performed by NimbleGen.

We used the limma package for bioconductor to fit linear models to the log transformed, normalized fluorescence intensities and calculated p-values for differential expression using empirical Bayesian methods [48]. These p-values were then used to calculate q-values with the Q-Value package for R [49]. All lists of differentially expressed genes were obtained by q-value thresholds (<.01, <.001). We calculated p-values for the chance of observing a given intersection of two gene lists, a and b, where b is the larger list, by summing over a hypergeometric distribution density function in R. This was performed using the function sum(dhyper(x, m, n, k)) where x represents a vector {i, i+1, i+2,..., j-1, j} (i being the number of genes in the intersection of a and b, and j being the number of genes in a), m is the number of gene in b, n = 15,634-m (i.e. the number of genes on the arrays but not in b), and k is the number of gene in a.

In order to determine the random occurrence of enriched GO-terms, we used our experimental expression array data set and randomized the gene names associated with expression values using the "sample" function in R, and used in the following context: sample(x, length(x), replace = FALSE, prob = NULL) where x is a vector of gene IDs. The GO-term analysis was performed on this randomized set as described below.

### RT-PCR quantitation

We designed primers specific for 8 genes that were misexpressed in both *sticky *and *dFmr1 *mutants (Additional File 4). RNA was prepared as above from females derived from different crosses than those used for the expression arrays. First strand cDNA synthesis was done using oligo-dT and Omniscript reverse transcriptase from Qiagen. Serial dilutions of cDNA were PCR amplified for *w*^1118^; +/+; *dFmr1*^3^/*dFmr1*^3 ^females and +/+; +/+; *sti*^3^/*sti*^*Z*3-5829 ^and Oregon R-S females. PCR products were electrophoresed on agarose gels and visualized with ethidium bromide. PCR products in the linear range were imaged and fluorescence was quantitated using ImageQuant v5.2 software. Local average background fluorescence was subtracted and the signal for each gene was normalized to a housekeeping gene (*gpdh*) that showed consistent expression between genotypes on the arrays. Up to ten replicates were done for each gene and p-values were calculated by Student's T-test.

### Genetic network analysis

Approximately 46,000 interactions (edges) between 9,196 genes (nodes) were downloaded from the Biomolecular Interaction Database (BIND) and the Database of interacting proteins (DIP) and integrated into Cytoscape using BioNetBuilder [50,51] to construct a protein-protein interaction network for *Drosophila melanogaster*. We were able to associate transcript fold changes and p-values with 8846 (>96%) of these genes. The jActiveModules plug-in [34] was used to find and score sub-networks based on the significance of their aggregate changes in expression. We searched for a single path adjusting score for size and using regional scoring with a search depth of 1 and max depth from start nodes of 2. We then identified biological process gene ontology terms that were significantly over represented (Benjamini Hochberg corrected p < .001) within the highest scoring sub-networks using the BiNGO plug-in [52].

### FlyAtlas

We downloaded the entire FlyAtlas annotated dataset from [35]. We then parsed out only the gene names and enrichment factors for each tissue (i.e. ratios of single tissue expression levels to whole fly expression). To construct lists with similar numbers of tissue enriched genes we had to determine an appropriate enrichment factor cutoff for each tissue. This was necessary because different tissues contribute different amounts of gene expression to the total observed in a whole fly. For each tissue, we plotted enrichment factors versus the total number of genes represented on the arrays that had an enrichment factors at least as great. We then fit power distributions (y = ax^b^) to these data and calculated the enrichment factor cutoffs predicted to provide the top 500 most enriched genes. These cutoffs had to be manually adjusted for ovaries and testes. We compared each tissue enriched gene list to each of our mutant misexpression lists (*sti*:q<.001, *sti*:q<.01, *dFmr1*:q<.001, and *dFmr1*:q<.01). We were then able to calculate p-values based on the number of genes present in both lists. This was performed by summing over the density of a hypergeometric distribution in R.

## Authors' contributions

CRB performed the RNA extractions and computational analyses, designed experiments, and participated in drafting the manuscript. AME collected oocyte polarity defect data. SJS performed the genetic interaction tests. DCZ designed experiments and participated in drafting the manuscript. GB collected oocyte polarity defect data, designed experiments, and participated in drafting the manuscript.

## Supplementary Material

Additional file 1 **Expression profiles for all genes in *sticky *mutants compared to wild-type**. For each gene, log fold changes are reported for *sticky *mutants compared to wild-type controls. Also included are moderated t-statistics (t), raw p-values, Benjamini Hochberg adjusted p-values, log odds scores (B), and q-values. Genes are sorted based on significance of expression changes. The table can be viewed in Microsoft Excel. Click here for file

Additional file 2 **Expression profiles for all genes in *dFmr1 *mutants compared to wild-type**. For each gene, log fold changes are reported for *dFmr1 *mutants compared to wild-type controls. Also included are moderated t-statistics (t), raw p-values, Benjamini Hochberg adjusted p-values, log odds scores (B), and q-values. Genes are sorted based on significance of expression changes. The table can be viewed in Microsoft Excel. Click here for file

Additional file 3 **List of genes commonly misexpressed in *sticky *and *dFmr1 *mutants with q<.01**. Log fold changes and p-values are reported for all genes that were misexpressed in both sticky and dFmr1 mutants with q<.01. The table can be viewed in Microsoft Excel. Click here for file

Additional file 4 **List of primers used for RT-PCR validation of microarray data**. For each transcript that was measured by RT-PCR, two primer sequences are provided. Click here for file
