# Supplementary material for: Genetic and systems level analysis of Drosophila sticky/citron kinase and dFmr1 mutants reveals common regulation of genetic networks
Source: BMC Syst Biol. 2008 Nov 25;2:101. doi: 10.1186/1752-0509-2-101 (PMC2610033; doi:10.1186/1752-0509-2-101)
Supplement: Additional file 4 — List of primers used for RT-PCR validation of microarray data. For each transcript that was measured by RT-PCR, two primer sequences are provided. [file 1752-0509-2-101-S4.pdf]

Gene Name	Amplicon Length	Direction	Sequence (5'-3')
gpdh	708	Forward	CCATGGTTAAAACAATACCAGGATGTACTG
		Reverse	GATAGCGATGGCATTTGGCTTAA
Cbl -L	291	Forward	TTGGATGAGGACATCGTTGA
		Reverse	ATAGAGCAGTGGCTGCGATT
Argk	757	Forward	TTCAAGAAGACCGACAAGCACCC
		Reverse	CATCGTACATCTCCTTGACGGCC
Tsf1	753	Forward	TGGCGGAGGAACCCATTTATC
		Reverse	CGAAATTCTCCGGATTGCCC
fh	749	Forward	CTAAAAAATGTTTGCCGGTCGTTTG
		Reverse	GCTTCACAGCTCCAGGGAAGTATAAGTATA
Reg-3	734	Forward	TGGTGCCCACCAAGCAGACGAA
		Reverse	ATCATGGCCGGTGGACAGGAGT
PGRP-SB1	542	Forward	CACATCAACGGCAATTAGTTTTGTG
		Reverse	TCTTGATCTCGTTGTACAGAGCATCAC
Ag5r2	765	Forward	TTGGGTGTTAATCGCAACAAAGG
		Reverse	GGATGTTGAGCAGAGGTTACCAAACTC
CG10659	603	Forward	AAAGACGGCATTACCATAAGGACCA
		Reverse	AAAGATCGGTCGTCCTTGATCGT
